# Reduction of SOST gene promotes bone formation through the Wnt/β‐catenin signalling pathway and compensates particle‐induced osteolysis

**DOI:** 10.1111/jcmm.15084

**Published:** 2020-03-05

**Authors:** Zai Hang Zhang, Xin Yu Jia, Jing Yi Fang, Hao Chai, Qun Huang, Chang She, Peng Jia, De Chun Geng, Wei Xu

**Affiliations:** ^1^ Department of Orthopedics The Second Affiliated Hospital of Soochow University Suzhou China; ^2^ The Experiment Center The Medical College of Soochow University Suzhou China; ^3^ Department of Orthopedics The Affiliated Zhangjiagang Hospital of Soochow University Suzhou China; ^4^ Department of Orthopedics The First Affiliated Hospital of Soochow University Suzhou China

**Keywords:** osteoblast, osteocyte, osteolysis, SOST/sclerostin, wear debris

## Abstract

The increase in bone resorption and/or the inhibition of bone regeneration caused by wear particles are the main causes of periprosthetic osteolysis. The SOST gene and Sclerostin, a protein synthesized by the SOST gene, are the characteristic marker of osteocytes and regulate bone formation and resorption. We aimed to verify whether the SOST gene was involved in osteolysis induced by titanium (Ti) particles and to investigate the effects of SOST reduction on osteolysis. The results showed osteolysis on the skull surface with an increase of sclerostin levels after treated with Ti particles. Similarly, sclerostin expression in MLO‐Y4 osteocytes increased when treated with Ti particles in vitro. After reduction of SOST, local bone mineral density and bone volume increased, while number of lytic pores on the skull surface decreased and the erodibility of the skull surface was compensated. Histological analyses revealed that SOST reduction increased significantly alkaline phosphatase‐ (ALP) and osterix‐positive expression on the skull surface which promoted bone formation. ALP activity and mineralization of MC3T3‐E1 cells also increased in vitro when SOST was silenced, even if treated with Ti particles. In addition, Ti particles decreased β‐catenin expression with an increase in sclerostin levels, in vivo and in vitro. Inversely, reduction of SOST expression increased β‐catenin expression. In summary, our results suggested that reduction of SOST gene can activate the Wnt/β‐catenin signalling pathway, promoting bone formation and compensated for bone loss induced by Ti particles. Thus, this study provided new perspectives in understanding the mechanisms of periprosthetic osteolysis.

## 1 INTRODUCTION

Artificial joint replacement is an effective method for the treatment of several joint diseases and senile femoral neck fractures. With increase in the ageing of population, the number of artificial joint replacements has also increased. Aseptic loosening of the implant caused by osteolysis induced by wear particles is a major complication of joint replacements.^1^ Wear debris produced by artificial joint can activate inflammatory cells, which release pro‐inflammatory cytokines that further stimulate osteoclast differentiation leading to bone resorption.^2^, ^3^, ^4^ On the other hand, wear debris directly suppresses osteoblasts functions and stimulates mediators that participate in the interaction between osteoblasts and osteoclasts, which leaded to a reduction in bone formation.^5^, ^6^, ^7^ In short, osteolysis surrounding prosthetic is a form of bone destruction and results in bone remodelling which increases bone resorption and might inhibit bone formation. However, the precise mechanism of periprosthetic osteolysis still remains unclear. Currently, there is no effective method to prevent and treat periprosthetic osteolysis.

Some studies have shown that osteocytes are the central regulators of cellular communication and bone remodelling which exchange information with osteoblasts and osteoclasts through tubules and dendrites.^8^, ^9^, ^10^, ^11^ Apoptosis of osteocytes increases osteoclast number and activity. Osteocytes express RANKL to induce osteoclast differentiation from precursor cells. Osteocyte activities may promote the mineralization of osteoblasts. Osteocyte regulation of osteoblasts and osteoclasts during bone remodelling can provide new perspectives in understanding the mechanisms involved in periprosthetic osteolysis.^12^, ^13^ Recently, Ormsby reported that osteocytes exposed to wear debris up‐regulated the expression of inflammatory cytokines, such as MMP‐13, IL‐6 and TNF‐α.^14^ However, research on whether osteocytes participate in periprosthetic osteolysis and how osteocytes control osteoblasts and osteoclasts in this process is still lacking. The SOST gene, which is expressed exclusively in osteocytes, has gained attention because of its role in regulating bone formation and resorption.^15^ Sclerostin, a protein synthesized by the SOST gene, inhibits the Wnt signalling pathway as it binds to a co‐receptor in the Wnt signalling pathway.^16^ Some studies have shown that SOST/sclerostin has a bidirectional regulation.^7^, ^17^ Sclerostin inhibits osteoblast differentiation and induces osteoblast apoptosis, meanwhile, stimulates osteocytes to secrete RANKL and activates osteoclasts. Some studies have shown that SOST gene and sclerostin are associated with sclerosis, fracture healing and osteoporosis.^18^, ^19^, ^20^, ^21^ Bone volume of osteoporosis is increased, and implant fixation is improved after SOST/sclerostin blockage.^22^ However, it is still unclear whether SOST/sclerostin is involved in periprosthetic osteolysis and whether reduction of SOST gene is effective in delaying osteolysis caused by wear particles.

This study aimed to verify whether SOST silencing can promote bone regeneration to compensate osteolysis induced by titanium particles in mouse skulls. Effects of SOST on osteocytes and osteoblasts during osteolysis were evaluated with in vivo and in vitro experiments. This study provided information on SOST/sclerostin as a target in compensating osteolysis induced by wear debris and delaying joint loosening surrounding the prosthesis.

## 2 MATERIALS AND METHODS

### 2.1 Preparation of titanium particles

Pure titanium (Ti) particles were obtained from Johnson Matthey. According to the manufacturer, the Ti particles had an average diameter of 5.34 μm and 90% of particles were <10.0 μm in size. When used in cell cultures and animal experiments, such particles have been shown to effectively mimic wear particles retrieved from periprosthetic tissue.

The particles were prepared as described by Chen et al^23^ The absence of endotoxins was confirmed using a commercial detection kit (Biowhittaker). The concentration of endotoxin‐free Ti particles was 100 mg/mL, and particles were stored at 4°C in PBS. The suspension of titanium particles was diluted to appropriate concentrations and used in cell and animal experiments.

### 2.2 Surgery procedure and application of retroviral vectors

All animal experiments were approved by the Ethics Committee of the Second Affiliated Hospital of Soochow University. Forty 8‐week‐old C57BL/6 female mice weighing between 20 and 25 g were used in the animal experiments. The mice were randomly divided into four groups: (a) control, (b) titanium (Ti), (c) Ti + SOST‐RNAi low concentration (Ti + L‐siRNA) and (d) Ti + SOST‐RNAi high concentration (Ti + H‐siRNA). All mice were injected with 50 μL of adeno‐associated viral vectors under the skin in the mid‐portion of the skull. However, the viral vectors injected in the mice of the L‐siRNA group carried 10^9^ copies of the SOST gene siRNA and the viral vectors injected in the mice of the H‐siRNA group carried 10^10^ copies of the SOST gene siRNA. After one week, the mice were anesthetized with an intraperitoneal injection of 50 mg/kg pentobarbital and a 10 mm sagittal incision was performed in the middle of the skull. The mice of the Ti, Ti + L‐siRNA and Ti + H‐siRNA group were injected with a 50 μL suspension of Ti particles and PBS (100 mg/mL) under the periosteum on the sagittal suture of the skull. The control group was injected with an equal volume of PBS. After two weeks, cranial specimens of mice were obtained for radiation, histology and immunohistochemistry testing.

### 2.3 Micro‐CT analysis

Cranial specimen (n = 5 per group) were fixed in 4% paraformaldehyde and were analysed with a Micro‐CT (Scanoco) set with 10 μm per layer. The X‐ray parameters were set at 70 kV and 114 μA. Micro‐CTs were taken of a circular region of interest (ROI of 3 mm in diameter) located at the middle of each calvaria, and the following data were collected: bone mineral density (BMD), number of pores, bone volume (BV) and bone volume/tissue volume (BV/TV).

### 2.4 Histological and immunohistochemical analysis

After soaked with formalin for 2 days, the cranial specimens (n = 5 per group) were decalcified in 10% ethylene diamine tetraacetic acid (EDTA, Sigma‐Aldrich) for one month. Then, the specimens were trimmed and the parietal and frontal bone of mice covered with Ti particles were mainly selected. Following anhydration and paraffin embedding, calvaria samples were sectioned at 5 μm for haematoxylin and eosin (H&E) staining. Images of the H&E staining results were obtained with a microscope at a magnification of 20× with the midline suture in its centre. Bone tissue surface area (BS mm^2^) and eroded bone surface area (ES mm^2^) were calculated with Image Pro‐Plus 6.0.

Immunohistochemistry staining was performed to determine the expression of the following proteins: sclerostin (Abcam, optimal dilutions: 1/30), ALP (Abcam, optimal dilutions: 1/350), β‐catenin (Abcam, optimal dilutions: 1/500) and osterix (Abcam, optimal dilutions: 1/300). Osteocytes were marked by sclerostin and β‐catenin and osteoblasts were marked by ALP and osterix. Firstly, we processed the calvaria samples with dewaxing, gradient hydration and antigen retrieval. Sections were then incubated with primary antibodies in the dark at 4°C for 12 hours. After which, sections were rinsed and incubated with a buffer containing secondary antibodies at room temperature for 35 minutes. Each section was photographed around midsagittal suture of calvaria under a microscope with 20× and 40× magnification. Cells stained brown colour were considered the immunoreactive positive cells. Two independent observers counted the positive stained cells in three sections of each group (n = 5) under a microscope with 20× magnification.

### 2.5 Cell cultures

MLO‐Y4 osteocytes and MC3T3‐E1 osteoblasts were obtained from the Chinese Academy of Sciences Cell Bank. MLO‐Y4 cells were cultured on collagen‐coated dishes (rat tail collagen type I, Sigma‐Aldrich) and maintained in modified essential medium (αMEM) supplemented with 10% foetal bovine serum (FBS, Gibco) and 1% penicillin and streptomycin in a 5% CO_2_ atmosphere at 37°C. Twenty‐four hours after seeding, the MLO‐Y4 cells were incubated with 1 mg/mL Ti particles, except for cells from the control group, which did not receive Ti particles. For all cells, fresh medium was supplied every three days. The day on which Ti particles were added to cells was designated as day 0.

The in vitro model for the co‐culture of osteocytes and osteoblasts was established using a Millicell 6‐Cell Culture Insert Plate (Millipore) comprised of a polyethylene terephthalate (PET) membrane perforated with 1 µm pores, as described by Fujita et al.^24^ This cell co‐culture allows osteocytes to be in contact with osteoblasts; thus, information can be transmitted through dendrites, and allows osteocytes and osteoblasts to be isolated for detection. Basal medium for co‐culture experiments consisted of α‐MEM supplemented with 10% FBS, 1% penicillin and 1% streptomycin. The osteogenic differentiation medium consisted of 10% FBS, 50 μg/mL ascorbic acid and 10 mmol/L β‐glycerophosphate (Sigma‐Aldrich).

**Figure 1:**
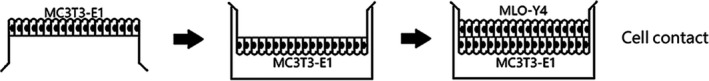
Cell co‐culture model: MC3T3‐E1 osteoblasts were seeded on the basal surface of the membrane, and MLO‐Y4 osteocytes were seeded on the apical surface of the membrane. Both cell types had direct contact with each other

In the cell co‐culture model, insert plates were inverted and the basal side of the membrane (bottom side of the insert) was seeded with 5 × 10^4^ MC3T3‐E1 osteoblasts in 500 μL of basal medium and incubated for 6 hours at 37°C to allow cellular adhesion. Inserts were then put into Millicell 6‐well tissue culture plates containing 1 mL of basal medium. Then, 5 × 10^4^ MLO‐Y4 osteocytes were seeded on the apical side of the membrane (top side of the insert) with 1 mL of basal medium and incubated overnight Figure 1. MLO‐Y4 cells on the upper side were treated with 1 mg/mL Ti particles diluted with basal medium, except for cells from the control group, which were not treated with Ti particles. The day on which Ti particles were added to cells was designated as day 0. The medium was replaced with osteogenic medium after three days, after which it was changed every three days.

### 2.6 SOST silencing and transfection

MLO‐Y4 osteocytes were silenced using short hairpin shRNA lentiviral particles (Sigma‐Aldrich), following the manufacturer's instructions. After searching the SOST gene sequence in GenBank, three interference sequences of SOST‐shRNA were designed, using the RNA interference from Invitrogen, and artificially synthesized by Sangon Biotech (China). Cells were injected with lentiviral particles carrying either scrambled or SOST‐ specific shRNA. The efficiency of shRNA was evaluated by measuring SOST mRNA and protein expression using real‐time PCR and Western blotting separately. Finally, the most effective sequence of SOST‐shRNA was selected and the shRNA sequence is 5′‐ GACAGCATATCTTACATTAAA ‐3′. MLO‐Y4 cells silenced for SOST were then used as SOST‐shRNA group in the following vitro experiments.

### 2.7 Western blot analysis for protein expression of sclerostin and active β‐catenin

MLO‐Y4 osteocytes were seeded into 6‐well plates and divided into three groups: control, Ti and Ti + SOST‐shRNA group. Cells in the SOST‐shRNA group underwent SOST silencing and transfection, while osteocytes of the control group underwent conventional culture for 48 hours. Cells of the Ti and Ti + SOST‐shRNA group were treated with 1 mg/mL Ti particles for 48 hours.

To detect the active protein of β‐catenin, the nuclear extraction of MLO‐Y4 cells was prepared using an NE‐PER Nuclear Cytoplasmic Extraction Reagent kit (Pierce) according to the manufacturer's instruction. Briefly, the treated cells were centrifuged and the cell pellet was suspended in cytoplasmic extraction reagent I and II, respectively, and then centrifuged. The insoluble pellet fraction, which contains crude nuclei, was resuspended in nuclear extraction reagent and incubated on ice, then centrifuged four times. The resulting supernatant, constituting the nuclear extract, was used for Western blot.

The collected cells for sclerostin detection were washed twice with PBS, treated with a lysis buffer and put on ice for 20 minutes, after which they were centrifuged at 15 000 *g* for 15 minutes. The supernatant was collected, and the protein concentration was determined using a BCA protein assay kit (Beyotime, China). Fifty micrograms of each sample were separated by 10%‐15% SDS‐PAGE and electro‐blotted onto PVDF membranes (The membranes were soaked in methanol for 3 minutes before western transfer). After blocking with 5% BSA (Sangon Biotech) for one hour at room temperature, membranes were incubated with a 1:1000 dilution of primary antibodies against sclerostin (Abcam) and β‐catenin (Cell Signaling Technology) overnight at 4°C. After washing three times with Tris‐buffered saline with Tween (TBST), membranes were incubated with horseradish peroxide (HRP) rabbit antimouse IgG for 60 minutes at room temperature. Samples were then washed with TBST three times, illuminated with electrochemiluminescence (ECL) and analysed using a GIS image analysis. As a loading control, anti‐β‐actin and anti‐Lamin A (Cell Signaling Technology) antibodies were used.

### 2.8 Immunofluorescence staining for the localization of active β‐catenin

To detect active β‐catenin in osteocytes, osteocytes were seeded into chamber slides. Cells were washed with PBS, fixed with cold PBS with 4% paraformaldehyde for 10 minutes, permeabilized with 0.1% Triton X‐100 for 5 minutes and then incubated in 5% BSA in 0.1% PBS‐Tween for one hour to block non‐specific protein‐protein interactions. The cells were then incubated with a rabbit antimouse β‐catenin primary antibody (Cell Signaling Technology) overnight at 4°C. Following three washes in PBS, cells were incubated with donkey anti‐rabbit IgG H&L Alexa Fluor^®^ 647 secondary antibody (Abcam) for an hour. Then, cells were washed in PBS and nuclei were stained with DAPI for 5 minutes. The slides were photographed using a ZEISS confocal microscope (ZEISS).

### 2.9 Alkaline phosphatase (ALP) activity and staining

The in vitro osteocyte‐osteoblast co‐culture model was also divided into three groups: control, Ti and Ti + SOST‐shRNA group. Cells of the control group underwent conventional culture, while osteocytes of the Ti and Ti + SOST‐shRNA group were treated with 1 mg/mL of Ti particles. ALP activity in the co‐culture supernatant was measured on day 7 after Ti particles were added. For such, medium was collected and centrifuged twice at 4000 *g* for 10 minutes in order to remove cell debris and Ti particles. ALP activity was evaluated using an Alkaline Phosphatase Assay Kit (Sigma‐Aldrich): assay mixtures contained 2‐amino‐2‐methyl‐1‐propanol, MgCl2, p‐nitrophenyl phosphate disodium and cell homogenates. After incubation, the reaction was stopped with NaOH and absorbance was read at 405 nm.

The cell co‐culture was maintained as described above. Similarly, ALP staining was performed on day 7 after Ti particles were added. Cells were washed three times with PBS prior to staining with an Alkaline Phosphatase Stain Kit: cells were fixed in methanol and overlaid with 5‐bromo‐4‐chloro‐3‐indolyl phosphate plus nitroblue tetrazolium chloride in Tris‐HCl, NaOH and MgCl2, followed by incubation at room temperature for two hours in the dark.

### 2.10 Mineralized nodule staining and detection of Ca^2+^ levels

The cell co‐culture was maintained as described above. Formation of calcified nodules was monitored with alizarin red S (Sigma‐Aldrich) staining on day 21 after Ti particles were added. For such, osteoblasts were washed with PBS, fixed with 70% ethanol and stained with 1% (w/v) alizarin red solution (pH 4.3) at room temperature. To quantify the amount of Ca^2+^ levels in alizarin red staining, the deposition was dissolved in 10% (w/v) cetylpyridinium chloride prepared in double‐distilled H_2_O (ddH_2_O) and quantified by measuring the OD value at 562 nm.

### 2.11 Statistical analysis

For each assay, three independent experiments (replicates) were performed. Data are expressed as the mean ± SD. One‐way analysis of variance (ANOVA) and post hoc multiple comparisons were used to determine differences among groups. Differences were considered significant when *P* < .05. All statistical analyses were performed with SPSS version 17.0.

## 3 RESULTS

### 3.1 Ti particles increased sclerostin expression in mice osteolysis model

**Figure 2:**
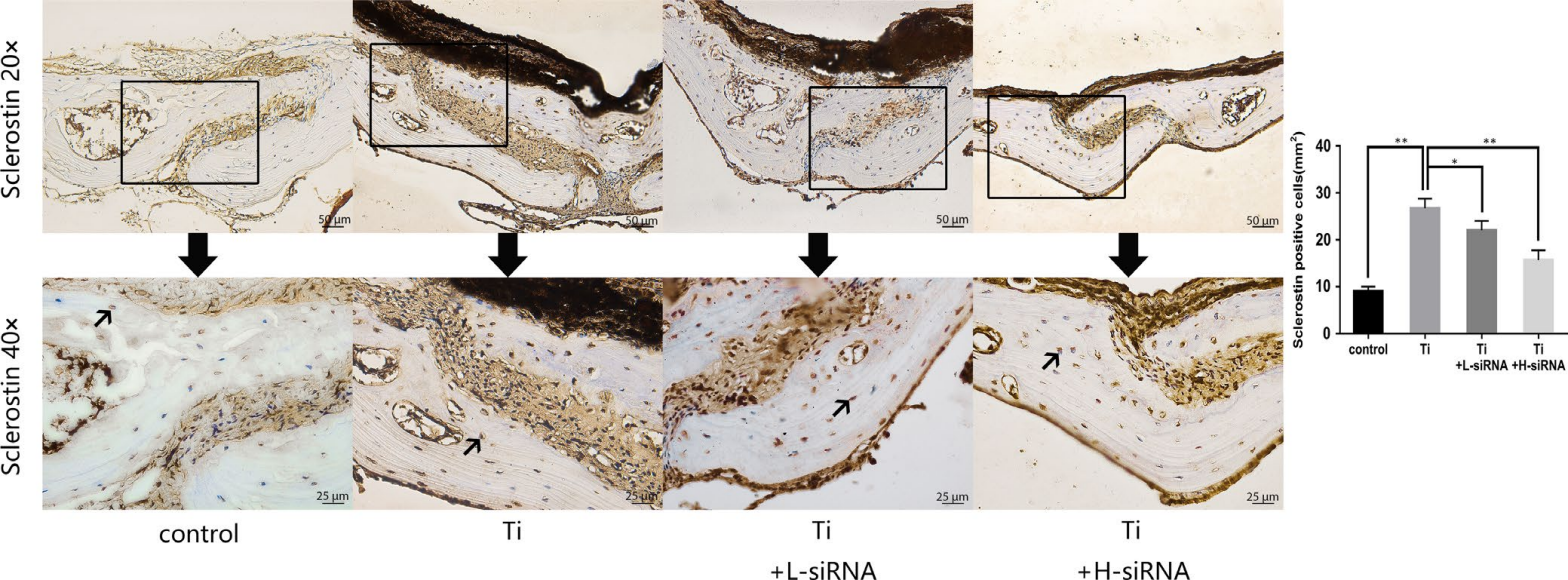
Immunohistochemical staining of sclerostin in mouse skulls and positive cell quantitative analysis. The images above were magnified 20 times. The images below were magnified 40 times which were the local area (boxed) of the images above. Black arrows in the images below indicated the sclerostin‐positive osteocytes which stained brown colour in the plate of calvaria. The expression of sclerostin in the Titanium (Ti) group was significantly higher in comparison to the control group (*P* < .01). After the interference of SOST, the expression of sclerostin in the Ti + L‐siRNA group and Ti + H‐siRNA groups was significantly lower than that in the Ti group (*P* < .05). Scale bar indicates 50 and 25 μm separately. *P < .05; **P < .01

Immunohistochemical staining was performed to determine changes in sclerostin expression. Sclerostin from the Ti group showed the highest expression. Conversely, sclerostin expression from the Ti + L‐siRNA and Ti + H‐siRNA group was significantly reduced, indicating that SOST siRNA carried by adeno‐associated viral vectors was effective to suppress sclerostin expression (Figure 2).

### 3.2 SOST reduction retarded bone loss induced by Ti particles in mice skull

**Figure 3:**
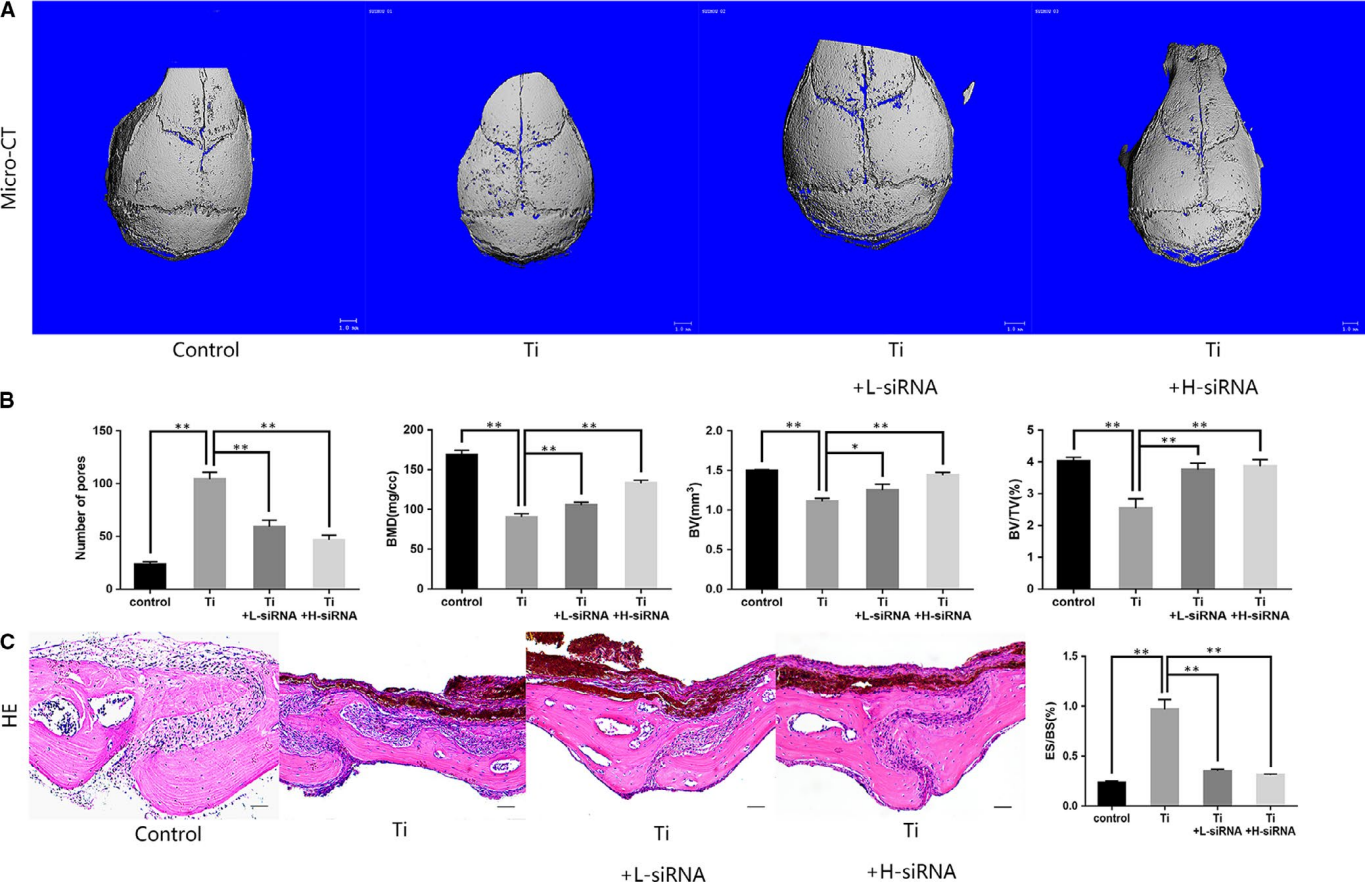
A, Micro‐CT images of calvaria in each experimental group showing that SOST reduction attenuated titanium‐induced osteolysis in a murine skull model. B, Number of pores, bone mineral density (BMD), bone volume (BV) and bone volume/tissue volume (BV/TV) observed in each experimental group. Data analysis revealed that Ti particles leaded to an increase in lytic pores and a decrease in BMD, BV and BV/TV in mice calvaria in comparison to the control (*P* < .01). Compared with the Ti group, the Ti + L‐siRNA and Ti + H‐siRNA group showed a decrease in lytic pores and an increase in BMD, BV and BV/TV (*P* < .05). C, Histological images of calvarial sections stained with H&E and the ratio of erosion surface to bone surface (ES/BS). HE staining showed that SOST gene reduction slowed the degree of osteolysis of mouse skulls. The ratio of ES/BS in the Ti group was far greater than the Control group (*P* < .01). The ratio of ES/BS in the Ti + L‐siRNA and Ti + H‐siRNA group was significantly lower in the comparison with the Ti group (*P* < .01). Scale bar indicates 50 μm. *P < .05; **P < .01

Skull surfaces of mice from the control group were smoother in comparison to skulls from the Ti group, which showed a higher degree of osteolysis. In contrast, the degree of osteolysis in the Ti + L‐siRNA and Ti + H‐siRNA group was significantly lower than that in the Ti group (Figure 3A). Quantitative analysis revealed that Ti particles lead to extensive lytic pores and decreased BMD, BV, and BV/TV in mice calvariae. Compared with the Ti group, the Ti + L‐siRNA and Ti + H‐siRNA group showed a reduction in the number of pores and an increase in BMD, BV and BV/TV (*P* < .05) (Figure 3B).

According to the H&E staining, the eroded surface observed in skulls from the Ti group was greater than in the Ti + L‐siRNA and Ti + H‐siRNA group (*P* < .05). Similarly, the ratio between the eroded surface (ES) and the bone surface (BS) in the Ti group was greater in comparison to the control group (*P* < .05). Correspondingly, the ratio of ES/BS in the Ti + L‐siRNA and Ti + H‐siRNA group was significantly lower in comparison to the Ti group (*P* < .05) (Figure 3C). Histological staining of mouse skulls showed the degree of bone erosion irritated by Ti particles was reduced after SOST reduction.

### 3.3 SOST reduction increased expression of osteoblastic markers in mice osteolysis model

**Figure 4:**
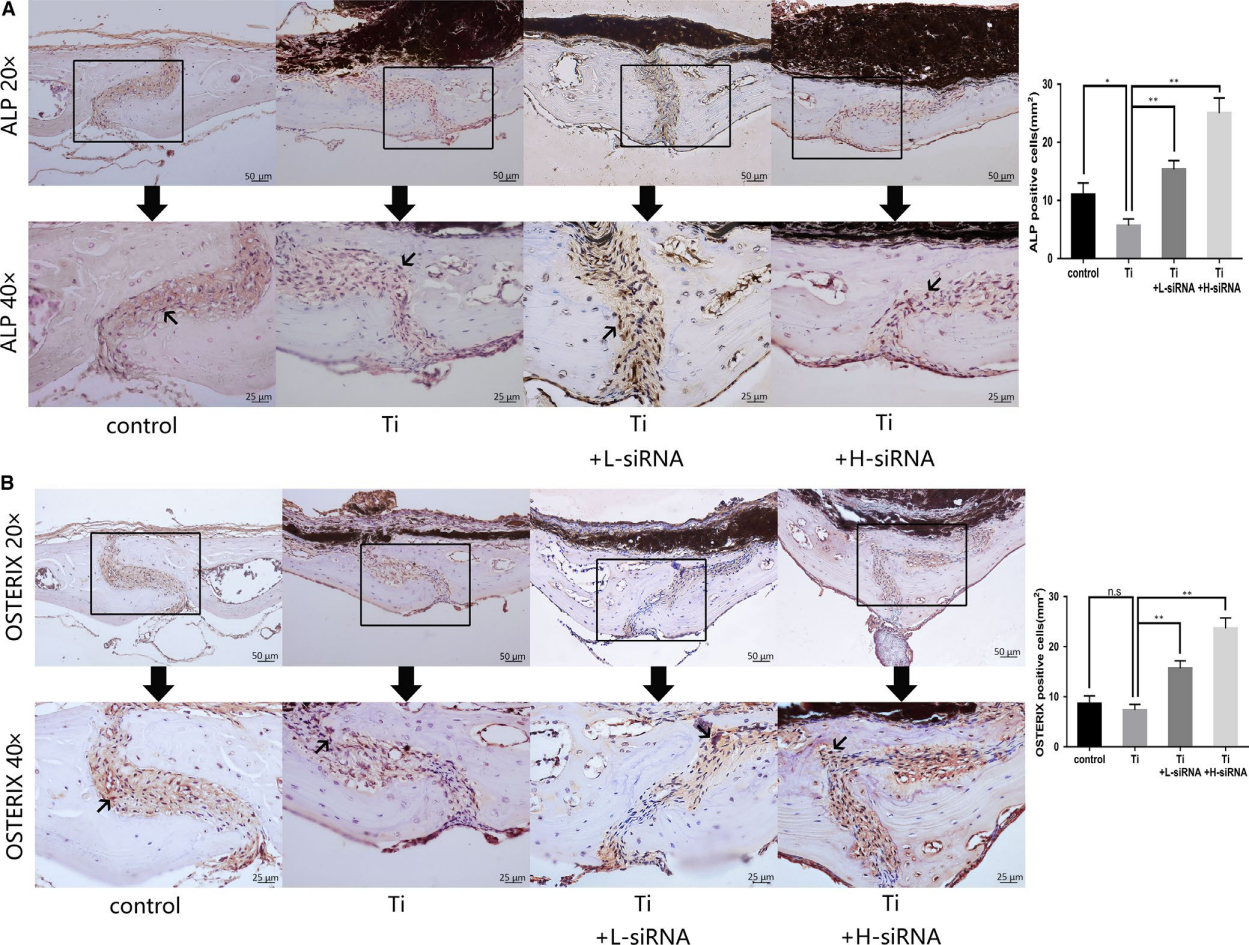
A, Alkaline phosphatase (ALP) immunohistochemical staining and quantification of ALP positive cell. The images above were magnified 20 times. The images below were magnified 40 times which were the local area (boxed) of the images above. Black arrows in the images below indicated ALP‐positive osteoblasts which stained brown colour around the suture of calvaria. Osteolysis induced by Ti leads to a significant decrease in ALP expression of cells compared to the control group (*P* < .05). The Ti + L‐siRNA and Ti + H‐siRNA groups had a significant improvement in the expression of ALP compared to the Ti group (*P* < .01). B, Osterix immunohistochemical staining and quantification of osterix‐positive cells. The images above were magnified 20 times. The images below were magnified 40 times which were the local area (boxed) of the images above. Black arrows in the images below indicated the Osterix‐positive osteoblasts which stained brown colour around the suture of calvaria. Osterix expression of cells from the Ti group was not significantly different in comparison to the control group. The Ti + L‐siRNA and Ti + H‐siRNA groups had a significant improvement in the expression of Osterix compared to the Ti group (*P* < .01).Scale bar indicates 50 and 25 μm separately. **P < .01; n.s: not significant

In order to probe into the effects of SOST expression on bone formation during osteolysis induced by wear particles, we examined changes in osteoblastic markers, ALP and osterix, using immunohistochemical staining. Our results showed that ALP expression in the Ti group showed a significant decrease compared to the control group (*P* < .05), while the expression of osterix in Ti group did not differ significantly from the control group. However, the expression of ALP and osterix in the Ti + L‐siRNA and Ti + H‐siRNA group was significantly greater compared to the Ti group (*P* < .05; Figure 4). Thus, SOST reduction might promote bone formation and compensate for bone loss induced by titanium particles.

### 3.4 SOST reduction increased expression of β‐catenin in mice osteolysis model

**Figure 5:**
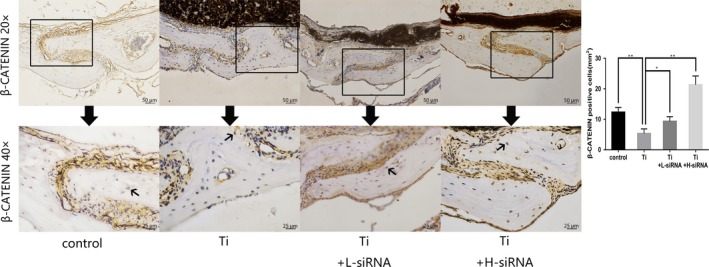
Immunohistochemical staining of β‐catenin and quantification of β‐catenin‐positive cells. The images above were magnified 20 times. The images below were magnified 40 times which were the local area (boxed) of the images above. Black arrows in the images below indicated the β‐catenin‐positive osteocytes which stained brown colour in the plate of calvaria. Osteolysis induced by Ti particles decreased the expression of β‐catenin (*P* < .01). After the suppression of SOST, the expression of β‐catenin was significantly increased in the Ti + L‐siRNA and Ti + H‐siRNA groups in comparison with the Ti group (*P* < .05). Scale bar indicates 50 μm and 25 μm separately. *P < .05; **P < .01

Since SOST gene was able to inhibit the Wnt/β‐catenin signalling pathway,^16^ we investigated the level of β‐catenin to further determine the mechanisms by which SOST reduction ameliorates titanium‐stimulated inhibition of bone formation. Osteolysis induced by titanium particles increased the expression of SOST, but decreased the expression of β‐catenin, while the expression of β‐catenin increased after SOST reduction. Despite exposure to Ti particles, expression of β‐catenin from the Ti + L‐siRNA and Ti + H‐siRNA group increased compared to that from the Ti group (Figure 5). Thus, SOST reduction might exert an osteogenic effect by activating the Wnt/β‐catenin signal cascade.

### 3.5 Ti particles increased SOST expression and decreased β‐catenin expression, and reduction of SOST improved β‐catenin expression in vitro

**Figure 6:**
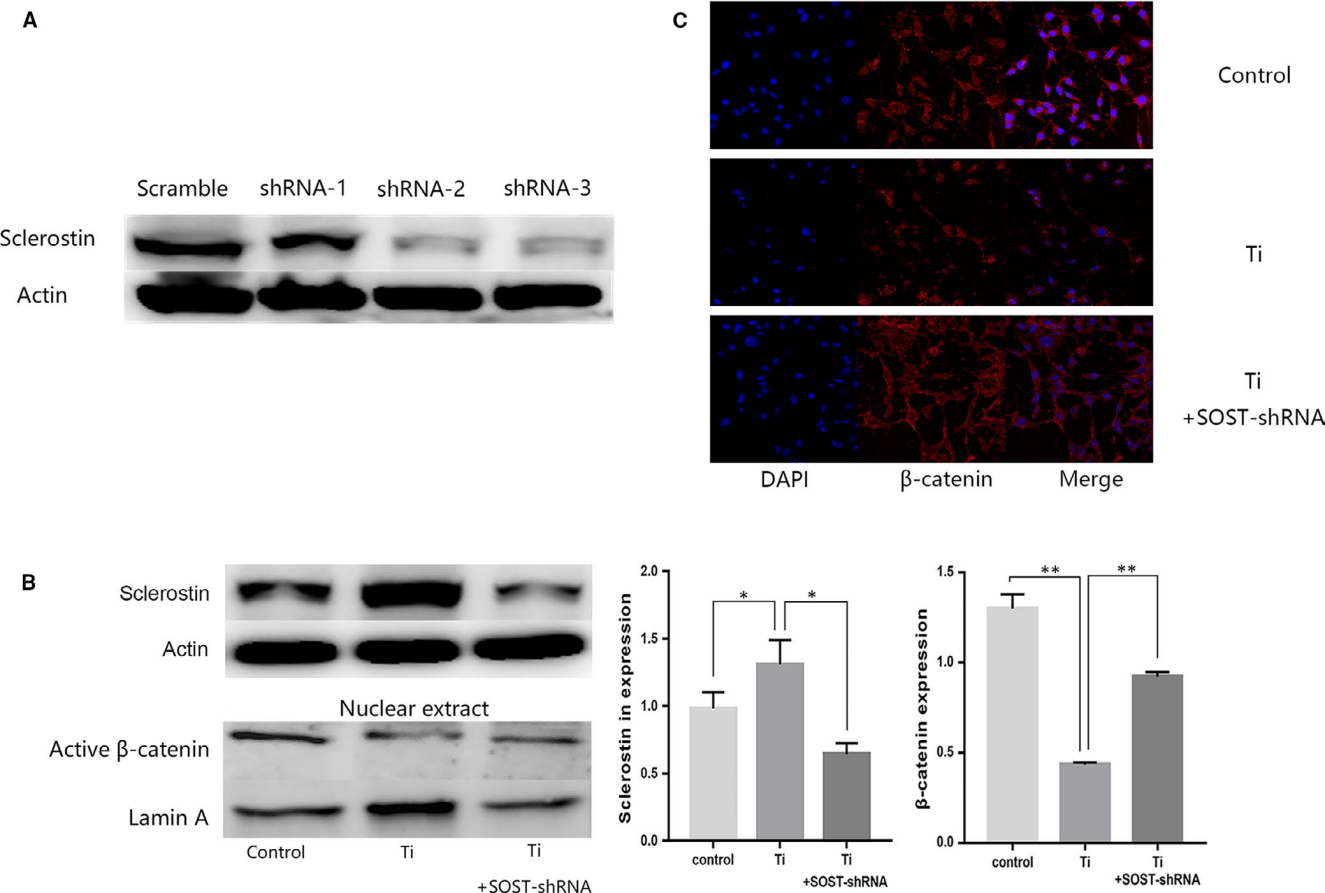
A, Cells were infected with lentiviral particles carrying either scrambled or SOST‐specific shRNA. The efficiency of shRNA was evaluated by measuring SOST protein expression using Western blotting. The second sequence of shRNA was chosen for experiments of SOST silence. B, Western blot of protein levels and data analysis of sclerostin and active β‐catenin after titanium interference. The in vitro osteocyte osteoblast co‐culture model was also divided into three groups: control, Ti group and Ti + SOST‐shRNA group. Ti particles at 48 h significantly increased SOST expression compared with the control (*P* < .05). Inversely, Ti particles decreased the expression level of active β‐catenin in nucleus (*P* < .05). After SOST silencing, the decrease of sclerostin expression in osteocytes leads to an increase in active β‐catenin expression levels, even though cells had been treated with Ti particles (*P* < .01). *P < .05, **P < .01. C, The distribution location of β‐catenin in osteocytes by immunofluorescence analysis. Blue, DAPI nuclear staining; Red, β‐catenin staining. Through immunofluorescence staining, the results showed that β‐catenin accumulated not only in the cytoplasm but also in nucleus of osteocytes. But β‐catenin localized in osteocytic nucleus decreased in Ti group compared with the control. After SOST silencing, more β‐catenin accumulated and translocated to the nucleus, even though cells had been treated with Ti particles

Since Ti particles induced osteolysis in the skull of mice with the increase of sclerostin in vivo, we further verified sclerostin expression of osteocytes in vitro. Forty‐eight hours after treating cells with 1 mg/mL of Ti particles, we observed a clear increase in sclerostin protein levels compared with the control (*P* < .05). Inversely, Ti particles decreased the protein of expression of active β‐catenin in osteocyte nucleus (*P* < .05). Through immunofluorescent staining, we found that β‐catenin in cell nucleus decreased in Ti group compared with the control (*P* < .05). We designed three interference sequences of SOST‐shRNA and verified them. The second and third shRNA sequences significantly reduced the expression of SOST, and we chose the most effective sequence, the second shRNA, for experiments of SOST silence. After SOST silencing, active β‐catenin protein levels increased and more β‐catenin were localized in cell nucleus, even though cells had been treated with Ti particles (*P* < .05; Figure 6). The change of sclerostin and β‐catenin expression in vitro were consistent with that found in the mice osteolysis model, suggesting that SOST might be involved in osteolysis induced by wear debris through the Wnt/β‐catenin signalling pathway.

### 3.6 Osteocyte change induced by Ti particles impaired the osteoblastic capacity and silencing SOST promoted osteoblast differentiation

**Figure 7:**
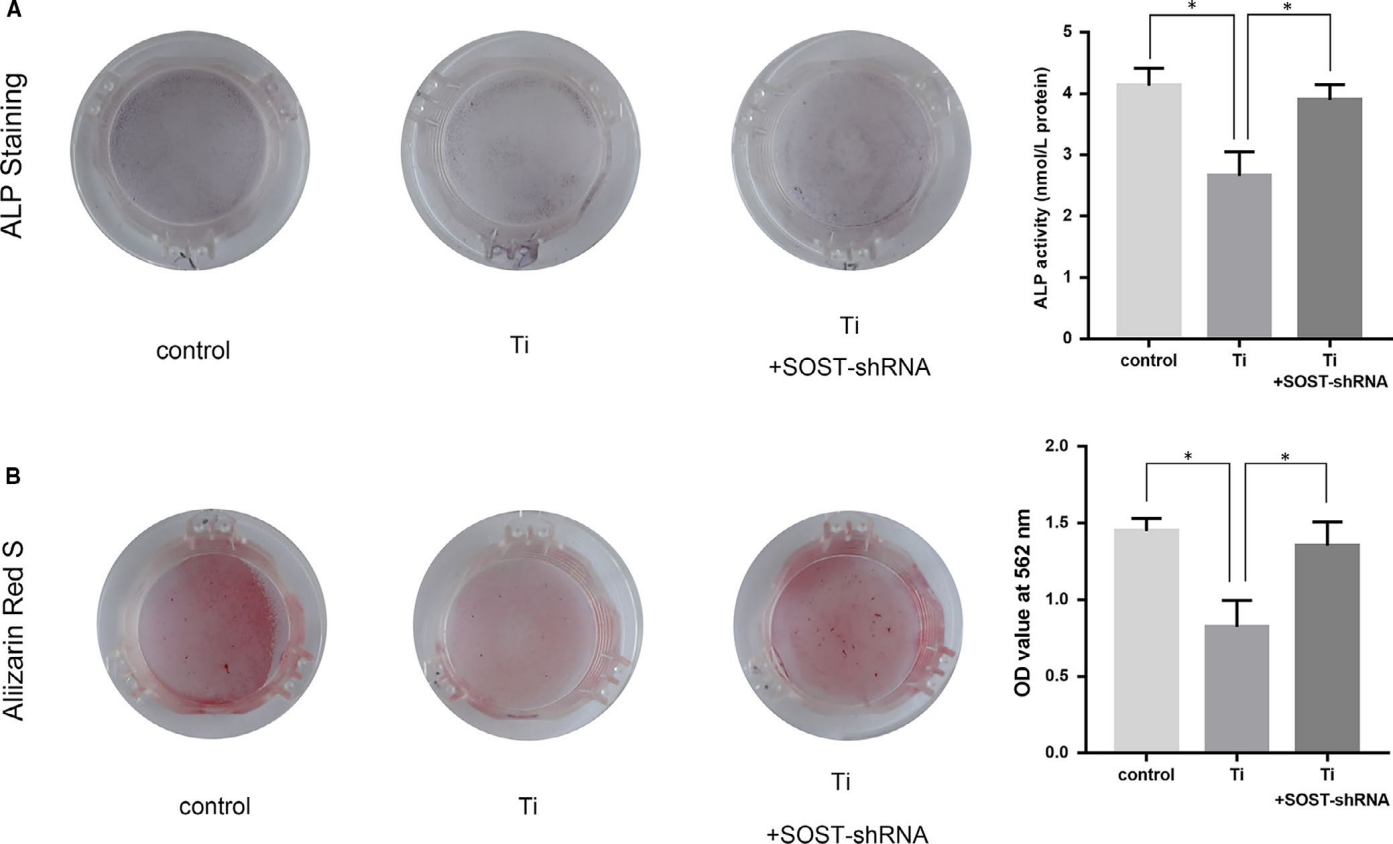
A, Alkaline phosphatase (ALP) staining results and data analysis of ALP activity on day 7 of the culture. The in vitro osteocyte‐osteoblast co‐culture model was also divided into three groups: control, Ti group, and Ti+SOST‐shRNA group. The number of ALP positive cells decreased and ALP activity of MC3T3‐E1 cells significantly decreased in the Ti group compared to the control group (*P* < .05). After SOST silencing, even though osteocytes had been treated with Ti particles, ALP positive cells increased and ALP activity of MC3T3‐E1 cells increased significantly in comparison with the Ti group (*P* < .05). B, Results and data analysis of mineralized nodules and calcium levels on day 21 of the culture. The number of mineralized nodules observed in MC3T3‐E1 cells decreased significantly in the cell co‐culture group treated with Ti particles. Calcium level in the cell co‐culture groups treated with Ti particles was lower significantly than the control (*P* < .05). After SOST silencing, even though osteocytes had been treated with Ti particles, the number of mineralized nodules increased and calcium level was higher significantly in comparison with the Ti group (*P* < .05). *P < .05

To determine the relationship of SOST and bone formation during osteolysis induced by Ti particles, we used an in vitro cell co‐culture model in which wear particles were only in contact with osteocytes but not with osteoblasts. Alterations in osteoblasts triggered by osteocyte titanium‐induced changes were recorded. As Figure 7A showed that ALP activity of MC3T3‐E1 osteoblast cells significantly decreased in the Ti group compared to the control group (*P* < .05). However, after SOST silencing, ALP activity of MC3T3‐E1 cells increased significantly in comparison with the Ti group (*P* < .05). Similarly, the number of mineralized nodules observed in MC3T3‐E1 cells decreased significantly in the cell co‐culture group that was treated with Ti particles. Calcium level in the group treated with Ti particles was lower significantly than the control (*P* < .05). The number of mineralized nodules and calcium levels increased significantly in SOST‐shRNA group in comparison with the Ti group (*P* < .05) (Figure 7B). These results indicated that titanium particles might induce osteocyte change, indirectly impairing the osteogenic capacity of osteoblasts, while silencing SOST in osteocytes can promote osteogenic differentiation.

## 4 DISCUSSION

Our results have shown that Ti wear particles induced osteolysis in mice skulls leading to an increase in sclerostin levels and that adding Ti particles to osteocytes in vitro also enhanced sclerostin levels. SOST reduction increased osteoblastic marker expression and promoted bone formation to compensate for particle‐irritated bone loss. Our evidence suggests that osteocytes and the characteristic marker, SOST, may play an important role in periprosthetic osteolysis.

Periprosthetic osteolysis, in which wear debris induces a biological reaction in histiocytes, is the most important factor in aseptic loosening. The abrasive particles generated by the friction of artificial joint parts activate inflammatory cells surrounding the prosthesis, such as macrophages, monocytes and fibroblasts. These inflammatory cells release various inflammatory substances, such as tumour necrosis factor‐α, interleukin 6, matrix metalloprotein‐2 and further activate osteoclasts that lead to the increase of bone resorption. On the other hand, inflammatory substances also inhibit osteoblast functions, which leads to a reduction in bone formation.^25^, ^26^ In other words, osteolysis is bone destruction which the abrasive particles initiated inflammatory reaction and results in the bone remodelling. The dynamic balance between bone formation and resorption is the basis for maintaining bone homeostasis. Theoretically, the inhibition of bone resorption and/or the increase in bone formation can alleviate or compensate osteolysis induced by wear particles.

Knowledge regarding the importance of osteocytes in the regulation of osteoblasts and osteoclasts has provided a new view on the mechanisms of periprosthetic osteolysis. Studies have reported that adding wear debris to osteocytes increased levels of inflammatory cytokines, leading to osteocyte apoptosis and osteoclast formation.^12^, ^13^, ^14^, ^27^ However, the regulation of injured osteocytes on osteoblasts during osteolysis as a result of wear debris remains largely unknown.

Meanwhile evidences have indicated that SOST/sclerostin, which are produced exclusively by osteocytes, play a key role in controlling bone formation and resorption.^28^, ^29^, ^30^ For instance, sclerostin inhibited bone formation as it prevented osteoblasts differentiation.^31^, ^32^, ^33^ Treatment with sclerostin antibody improved implant fixation by increasing bone‐implant contact and trabecular bone volume and architecture.^19^ Additionally, the sclerostin antibody stimulated bone formation and inhibited bone resorption in a rat model.^22^ Sclerostin antibodies increased cortical bone volume and improved bone strength in ovariectomized rat model.^34^ Therefore, our study aimed to confirm whether SOST/sclerostin was involved in periprosthetic osteolysis. As expected, we observed extensive lytic pores on the surface of the calvarial bone and an increase of sclerostin after Ti particles were injected. By contrast, histological analyses revealed that SOST reduction decreased the bone erosion and made the surface of the calvaria smoother. Similarly, a lower number of pores and a greater BMD and BV values further confirmed that the osteolytic action was compensated when the SOST gene was silenced, suggesting that SOST reduction exerts a protective effect on osteolysis induced by Ti particles.

Osteogenic bone formation is the most common event of bone remodelling. Suppression of bone formation plays an important role in developing prosthetic loosening, and promotion of bone formation is a promising target to control such osteolytic diseases. Consequently, we investigated the effects SOST reduction on bone formation. As illustrated by the histological results, suppression of SOST increased in MBD and the number of ALP‐ and osterix‐positive cells in vivo. It has been widely recognized that ALP and osterix are the characteristic markers of osteoblast differentiation 35, 36, 37, 38; thus, these findings indicate that osteoblast is the target of SOST and that SOST reduction therapy enhances bone regeneration in osteolytic calvaria irritated by Ti particles.

As a characteristic marker of osteocyte, SOST/sclerostin was confirmed to be involved in periprosthetic osteolysis. This suggests that osteocytes play an important role in osteolysis induced by Ti particles. To further verify the results in the mice skull model, we investigated the interaction between Ti particles and MLO‐Y4 osteocytes in vitro. Sclerostin expression increased with the addition of Ti particles, consistent with the changes of sclerostin levels observed in vivo. Furthermore, we co‐cultured MC3T3‐E1 osteoblasts with osteocytes such that osteocytes and osteoblasts were seeded on two sides of the same porous membrane, allowing cells to have direct contact through pores that simulate information exchange between osteocyte and osteoblast via dendritic processes. However, MC3T3‐E1 osteoblasts were not allowed to contact with the Ti particles, thus avoiding the direct effects of Ti particles on osteoblast. Our co‐culture results showed that ALP activity and mineralization of MC3T3‐E1 were suppressed when MLO‐Y4 cells were treated with Ti particles in the co‐culture model. Nonetheless, after SOST was silenced, ALP activity and mineralization of MC3T3‐E1 cells increased, even when cells were treated with Ti particles. This could indicate that Ti particles lead to osteocyte injury with an increase in expression of SOST/sclerostin, causing osteoblast dysfunction. Thus, this may explain how SOST reduction promotes bone formation, delaying bone loss in murine skull surfaces.

Wnt/β‐catenin signalling pathway is closely implicated in osteoblastic differentiation and mineralization.^39^, ^40^ When Wnt/β‐catenin signalling pathway is activated, β‐catenin is dephosphorylated and restore active. Active β‐catenin accumulates and translocates to the nucleus to activate target genes. SOST/sclerostin is strongly associated with bone formation via inhibition of canonical Wnt signalling. In our study, we observed that Ti particles increased significantly sclerostin expression while active β‐catenin expression decreased. Inversely, SOST reduction attenuated Ti particle‐induced suppression of bone formation with increasing β‐catenin levels, suggesting that the Wnt/β‐catenin signalling pathway is important in periprosthetic osteolysis. These results suggest that the SOST reduction activated the Wnt/β‐catenin signalling pathway, promoting bone formation and compensated for the bone loss induced by wear debris.

Previous studies have shown that sclerostin antibodies can be used to treat osteoporosis and skeletal abnormalities, increasing bone formation, bone mass and bone strength.^18^, ^19^, ^20^, ^21^, ^22^, ^41^, ^42^, ^43^ Additionally, sclerostin antibodies have been reported to promote fracture callus formation and increase the stability of internal fixation.^44^ However, studies addressing the role of sclerostin in osteolysis of prosthesis are still lacking. Our study demonstrated how osteolysis induced by Ti particles could be compensated by promoting bone formation through SOST silencing and its related mechanisms.

However, although our current findings suggest that SOST reduction has a positive effect on bone formation, there are still several shortcomings in our study. First, the murine skull osteolysis model is exactly not the same as joint replacement surgery. The model does not completely simulate joints (ie fluid pressure and mechanical load are different); thus, we were unable to perform biomechanical experiments, which need to be tested in order to improve biomechanical research. Finally, this experiment was mainly interested in the related effects of the SOST gene on osteocytes and osteoblasts, and mechanisms involving bone resorption and osteoclasts activity will be explored in future experiments.

## 5 CONCLUSION

In summary, we demonstrated that SOST gene reduction promoted bone formation and alleviated bone loss caused by Ti particles. These effects might be mediated by activating the Wnt/β‐catenin signalling pathway and enhancing osteogenic differentiation. Our results provided some information for the treatment of osteolysis surrounding prosthesis.

## CONFLICT OF INTEREST

The authors declare no conflict of interest.

## AUTHORS CONTRIBUTION

DCG and WX involved in study design. XYJ, ZHZ, JYF, HC and QH involved in study conduct. ZHZ, XYJ, JYF and QH involved in data collection. ZHZ, XYJ, JYF, CS, PJ and WX involved in data analysis. ZHZ, XYJ, JYF, DCG and WX involved in data interpretation. XYJ and ZHZ drafted the manuscript. ZHZ, DCG and WX involved in revising manuscript content.

## Data Availability

The authors confirm that the data supporting the findings of this study are available within the article.

## References

[1] Hallab NJ , Jacobs JJ . Biologic effects of implant debris. Bull NYU Hosp Jt Dis. 2009;67:182‐188. 19583551

[2] Laquerriere P , Grandjean‐Laquerriere A , Addadi‐Rebbah S , et al. MMP‐2, MMP‐9 and their inhibitors TIMP‐2 and TIMP‐1 production by human monocytes in vitro in the presence of different forms of hydroxyapatite particles. Biomaterials. 2004;25:2515‐2524. 14751736 10.1016/j.biomaterials.2003.09.034

[3] Lovric V , Goldberg MJ , Heuberer PR , et al. Suture wear particles cause a significant inflammatory response in a murine synovial airpouch model. J Orthop Surg Res. 2018;13:311‐318. 30522505 10.1186/s13018-018-1026-4 PMC6282382

[4] Shimizu S , Okuda N , Kato N , et al. Osteopontin deficiency impairs wear debris‐induced osteolysis via regulation of cytokine secretion from murine macrophages. Arthritis Rheum. 2010;62(5):1329‐1337. 20155835 10.1002/art.27400

[5] Lohmann CH , Dean DD , Köster G , et al. Ceramic and PMMA particles differentially affect osteoblast phenotype. Biomaterials. 2002;22:1855‐1863. 10.1016/s0142-9612(01)00312-x 11950056

[6] Chiu R , Ma T , Smith RL , Goodman SB . Ultrahigh molecular weight polyethylene wear debris inhibits osteoprogenitor proliferation and differentiation in vitro. J Biomed Mater Res. 2009;89:242‐247. 10.1002/jbm.a.32001 18442106

[7] Nakashima T , Hayashi M , Fukunaga T , et al. Evidence for osteocyte regulation of bone homeostasis through RANKL expression. Nat Med. 2011;17:1231‐1234. 21909105 10.1038/nm.2452

[8] Prideaux M , Findlay DM , Atkins GJ . Osteocytes: The master cells in bone remodelling. Curr Opin Pharmacol. 2016;28:24‐30. 26927500 10.1016/j.coph.2016.02.003

[9] Chen H , Senda T , Kubo KY . The osteocyte plays multiple roles in bone remodeling and mineral homeostasis. Med Mol Morphol. 2015;48:61‐68. 25791218 10.1007/s00795-015-0099-y

[10] Niedzwiedzki T , Filipowska J . Bone remodeling in the context of cellular and systemic regulation: the role of osteocytes and the nervous system. J Mol Endocrinol. 2015;55:R23‐R36. 26307562 10.1530/JME-15-0067

[11] Sims NA , Vrahnas C . Regulation of cortical and trabecular bone mass by communication between osteoblasts, osteocytes and osteoclasts. Arch Biochem Biophys. 2014;561:22‐28. 24875146 10.1016/j.abb.2014.05.015

[12] Kanaji A , Caicedo MS , Virdi AS , Sumner DR , Hallab NJ , Sena K . Co‐Cr‐Mo alloy particles induce tumor necrosis factor alpha production in MLO‐Y4 osteocytes: a role for osteocytes in particle‐induced inflammation. Bone. 2009;45:528‐533. 19497395 10.1016/j.bone.2009.05.020 PMC2725206

[13] Zhang Y , Yan M , Yu A , et al. Inhibitory effects of β‐tricalcium phosphate wear particles on osteocytes via apoptotic response and Akt inactivation. Toxicology. 2012;297:57‐67. 22522029 10.1016/j.tox.2012.04.002

[14] Ormsby RT , Solomon LB , Yang D , et al. Osteocytes respond to particles of clinically‐relevant conventional and cross‐linked polyethylene and metal alloys by up‐regulation of resorptive and inflammatory pathways. Acta Biomater. 2019;87:296‐306. 30690207 10.1016/j.actbio.2019.01.047

[15] Han Y , You X , Xing W , Zhang Z , Zou W . Paracrine and endocrine actions of bone‐the functions of secretory proteins from osteoblasts, osteocytes, and osteoclasts. Bone Res. 2018;6:16. 29844945 10.1038/s41413-018-0019-6 PMC5967329

[16] ten Dijke P , Krause C , de Gorter DJJ , Löwik CWGM , van Bezooijen RL . Osteocyte‐derived sclerostin inhibits bone formation: its role in bone morphogenetic protein and Wnt signaling. J Bone Joint Surg Am. 2008;90(Suppl 1):31‐35. 10.2106/JBJS.G.01183 18292354

[17] Sapir‐Koren R , Livshits G . Osteocyte control of bone remodeling: is sclerostin a key molecular coordinator of the balanced bone resorption–formation cycles? Osteoporos Int. 2014;25:2685‐2700. 25030653 10.1007/s00198-014-2808-0

[18] McClung MR , Grauer A , Boonen S , et al. Romosozumab in postmenopausal women with low bone mineral density. N Engl J Med. 2014;370:412‐420. 24382002 10.1056/NEJMoa1305224

[19] Virdi AS , Irish J , Sena K , et al. Sclerostin antibody treatment improves implant fixation in a model of severe osteoporosis. J Bone Joint Surg Am. 2015;97:133‐140. 25609440 10.2106/JBJS.N.00654

[20] Ominsky MS , Boyce RW , Li X , Ke HZ . Effects of sclerostin antibodies in animal models of osteoporosis. Bone. 2017;96:63‐75. 27789417 10.1016/j.bone.2016.10.019

[21] Jacobsen CM . Application of anti‐Sclerostin therapy in non‐osteoporosis disease models. Bone. 2017;96:18‐23. 27780792 10.1016/j.bone.2016.10.018 PMC5328800

[22] Virdi AS , Liu M , Sena K , et al. Sclerostin antibody increases bone volume and enhances implant fixation in a rat model. J Bone Joint Surg Am. 2012;94:1670‐1680. 22992878 10.2106/JBJS.K.00344 PMC3444952

[23] Chen M , Chen P‐M , Dong Q‐R , Huang Q , She C , Xu W . p38 signaling in titanium particle‐induced MMP‐2 secretion and activation in differentiating MC3T3‐E1 cells. J Biomed Mater Res A. 2014;102:2824‐2832. 24115593 10.1002/jbm.a.34956

[24] Fujita K , Xing Q , Khosla S , Monroe DG . Mutual enhancement of differentiation of osteoblasts and osteocytes occurs through direct cell‐cell contact. J Cell Biochem. 2014;115:2039‐2044. 25043105 10.1002/jcb.24880 PMC4169216

[25] Holt G , Murnaghan C , Reilly J , Meek RM . The biology of aseptic osteolysis. Clin Orthop Relat Res. 2007;460:240‐252. 17620815 10.1097/BLO.0b013e31804b4147

[26] Lange T , Schilling AF , Peters F , et al. Proinflammatory and osteoclastogenic effects of beta‐tricalciumphosphate and hydroxyapatite particles on human mononuclear cells in vitro. Biomaterials. 2009;30:5312‐5318. 19577291 10.1016/j.biomaterials.2009.06.023

[27] Ormsby RT , Cantley M , Kogawa M , et al. Evidence that osteocyte perilacunar remodelling contributes to polyethylene wear particle induced osteolysis. Acta Biomater. 2016;33:242‐251. 26796208 10.1016/j.actbio.2016.01.016

[28] Poole KES , van Bezooijen RL , Loveridge N , et al. Sclerostin is a delayed secreted product of osteocytes that inhibits bone formation. FASEB J. 2005;19:1842‐1844. 16123173 10.1096/fj.05-4221fje

[29] Wijenayaka AR , Kogawa M , Lim HP , Bonewald LF , Findlay DM , Atkins GJ . Sclerostin stimulates osteocyte support of osteoclast activity by a RANKL‐dependent pathway. PLoS ONE. 2011;6:e25900. 21991382 10.1371/journal.pone.0025900 PMC3186800

[30] Liu S , Virdi AS , Sena K , Sumner DR . Sclerostin antibody prevents particle‐induced implant loosening by stimulating bone formation and inhibiting bone resorption in a rat model. Arthritis Rheum. 2012;64:4012‐4020. 23192793 10.1002/art.37697

[31] Sutherland MK , Geoghegan JC , Yu C , Winkler DG , Latham JA . Unique regulation of SOST, the sclerosteosis gene, by BMPs and steroid hormones in human osteoblasts. Bone. 2004;35:448‐454. 15268896 10.1016/j.bone.2004.04.019

[32] Li F , Cain JD , Tombran‐Tink J , Niyibizi C . Pigment epithelium derived factor regulates human Sost/Sclerostin and other osteocyte gene expression via the receptor and induction of Erk/GSK‐3beta/beta‐catenin signaling. Biochim Biophys Acta Mol Basis Dis. 2018;1864:3449‐3458. 30076958 10.1016/j.bbadis.2018.07.034 PMC6176723

[33] Ueda M , Kuroishi KN , Gunjigake KK , Ikeda E , Kawamoto T . Expression of SOST /sclerostin in compressed periodontal ligament cells. J Dent Sci. 2016;11:272‐278. 30894984 10.1016/j.jds.2016.02.006 PMC6395252

[34] Ma YL , Hamang M , Lucchesi J , et al. Time course of disassociation of bone formation signals with bone mass and bone strength in sclerostin antibody treated ovariectomized rats. Bone. 2017;97:20‐28. 27939957 10.1016/j.bone.2016.12.003

[35] Fukayama S , Tashjian AH Jr . Involvement of alkaline phosphatase in the modulation of receptor signaling in osteoblast: Evidence for a difference between human parathyroid hormone‐related protein and human parathyroid hormone. J Cell Physiol. 1994;158:391‐397. 8126063 10.1002/jcp.1041580302

[36] Torii Y , Hitomi K , Yamagishi Y , Tsukagoshi N . Demonstration of alkaline phosphatase participation in the mineralization of osteoblasts by antisense RNA approach. Cell Biol Int. 1996;20:459‐464. 8931312 10.1006/cbir.1996.0060

[37] Matsubara T , Kida K , Yamaguchi A , et al. BMP2 regulates osterix through Msx2 and Runx2 during osteoblast differentiation. J Bio Chem. 2008;283:29119‐29125. 18703512 10.1074/jbc.M801774200 PMC2662012

[38] Leong W , Zhou T , Lim G , Li B . Protein palmitoylation regulates osteoblast differentiation through BMP‐induced osterix expression. PLoS ONE. 2009;4:e4135. 19125191 10.1371/journal.pone.0004135 PMC2607547

[39] Duan P , Bonewald LF . The role of the wnt/β‐catenin signaling pathway in formation and maintenance of bone and teeth. Int J Biochem Cell Biol. 2016;77:23‐29. 27210503 10.1016/j.biocel.2016.05.015 PMC4958569

[40] Agholme F , Aspenberg P . Wnt signaling and orthopedics, an overview. Acta Orthop. 2011;82:125‐130. 21438671 10.3109/17453674.2011.572252 PMC3235279

[41] Schoeman MA , Moester MJ , Oostlander AE , et al. Inhibition of GSK3 stimulates BMP signaling and decreases SOST expression which results in enhanced osteoblast differentiation. J Cell Biochem. 2015;116:2938‐2946. 26095393 10.1002/jcb.25241

[42] Li X , Ominsky MS , Warmington KS , et al. Sclerostin antibody treatment increases bone formation, bone mass, and bone strength in a rat model of postmenopausal osteoporosis. J Bone Miner Res. 2009;24:578‐588. 19049336 10.1359/jbmr.081206

[43] Williams DK , Parham SG , Schryver E , et al. Sclerostin antibody treatment stimulates bone formation to normalize bone mass in male down syndrome mice. JBMR Plus. 2017;2:47‐54. 30283889 10.1002/jbm4.10025 PMC6124205

[44] Morse A , Yu N , Peacock L , et al. Endochondral fracture healing with external fixation in the Sost knockout mouse results in earlier fibrocartilage callus removal and increased bone volume fraction and strength. Bone. 2015;71:155‐163. 25445453 10.1016/j.bone.2014.10.018

